# Biomodification of a Class-V Restorative Material by Incorporation of Bioactive Agents

**DOI:** 10.3390/dj7040110

**Published:** 2019-11-29

**Authors:** Tahani Binaljadm, Robert Moorehead, Thafar Almela, Kirsty Franklin, Lobat Tayebi, Keyvan Moharamzadeh

**Affiliations:** 1 School of Clinical Dentistry, University of Sheffield, Sheffield S10 2TA, UK; tmbinaljadm1@sheffield.ac.uk (T.B.); r.moorehead@sheffield.ac.uk (R.M.); tkdalmela@gmail.com (T.A.); K.L.Franklin@sheffield.ac.uk (K.F.); 2 School of Dentistry, Taibah University, Medina 42353, Saudi Arabia; 3 College of Dentistry, University of Mosul, Mosul 41002, Iraq; 4 School of Dentistry, Marquette University, Milwaukee, WI 53233, USA; lobat.tayebi@marquette.edu

**Keywords:** dental materials, glass ionomer cement, biocompatibility, oral mucosa, biomodification

## Abstract

Restoring subgingival class-V cavities successfully, demand special biological properties from a restorative material. This study aimed to assess the effects of incorporating bioactive materials to glass ionomer cement (GIC) on its mechanical and biological properties. Hydroxyapatite, chitosan, chondroitin sulphate, bioglass, gelatine and processed bovine dentin were incorporated into a GIC restorative material. Compressive strength, biaxial flexural strength (BFS), hardness, setting and working time measurements were investigated. Biocompatibility of the new materials was assessed using both monolayer cell cultures of normal oral fibroblasts (NOF) and TR146 keratinocytes, and a 3D-tissue engineered human oral mucosa model (3D-OMM) using presto-blue tissue viability assay and histological examination. Significant reduction in the compressive strength and BFS of gelatine-modified discs was observed, while chondroitin sulphate-modified discs had reduced BFS only (*p* value > 0.05). For hardness, working and setting times, only bioglass caused significant increase in the working time. NOF viability was significantly increased when exposed to GIC-modified with bovine dentine, bioglass and chitosan. Histological examination showed curling and growth of the epithelial layer toward the disc space, except for the GIC modified with gelatine. This study has highlighted the potential for clinical application of the modified GICs with hydroxyapatite, chitosan, bioglass and bovine dentine in subgingival class-V restorations.

## 1. Introduction

Root caries has become a significant dental problem in older adult population due to increase in age and retention of teeth with exposed root surfaces [1,2]. Several studies have demonstrated high failure rates of approximately 27.8% in 5 years and 36–87% in 13 years for class V restorations using currently available dental materials [3,4,5]. Systematic reviews indicated significant influence of the type of material and the adhesive system on the survival of restorations of non-carious cervical lesions [6,7]. Other reported prognostic factors include individual clinician, size of the cavity, cavity preparation technique, moisture contamination, patient age, presence or absence of enamel and caries. Predominant modes of failure for class V restorations includes loss of retention (83%) need for crowning (5%), facture (4%), partial loss of restoration (4%) and secondary caries (3%) [3].

Improving restorative materials for cervical class V cavities can be challenging due to different biomechanical requirements for the material compared to those for occlusal cavities. Stress generated in class V lesions due to occlusal loads can contribute to fracture and debonding of the restorative materials [8]. Materials such as resin modified glass ionomer cements (RMGICs), compomers and composite resins show promise as alternative materials to dental amalgams for occlusal cavities [9]. Nevertheless, these materials suffer from the inherent problem of polymerisation shrinkage, which is known to be a significant contributor to compromising the bond to enamel and more particularly dentin [10]. In addition, their placement is complicated by the need to use separate adhesive procedures and/or incremental placement techniques. Furthermore, safety issues have been raised with regards to unpolymerized toxic monomers released from resin-based restorative materials [11,12] and the ability of periodontal soft-tissues to attach to the surface of resin-based materials is significantly compromised. In vitro studies have shown human gingival fibroblasts were unable to attach onto the fresh surface of set conventional restorative materials [13]. However, BHK-21 fibroblast cell line was able to attach to the surface of GIC materials impregnated with bioactive glass and collage [14]. Periodontal ligament (PDL) fibroblasts were also able to attach onto the surface of set mineral trioxide aggregate (MTA) material [15]. MTA’s use as a class V restorative material is limited due to its long setting-time and susceptibility to wash-out as well as its poor aesthetic properties. Glass ionomer cement (GIC) is the only tooth-coloured bulk placement, intrinsically adhesive [16] restorative material with several other favourable properties including biocompatibility [17], fluoride release [18] and a coefficient of thermal expansion compatible with tooth tissues [19]. However, they lack the ideal flexural strength and wear resistance to perform satisfactorily in class V lesions [20].

The GICs available today have changed little since the time they were first introduced in 1978 as alumino-silicate poly acrylic acid cement (ASPA). Although systematic reviews suggest glass ionomer derivatives still have better retention rates in cervical restorations compared to self-etching adhesive systems, an enhanced glass ionomer-based restorative material with improved biological properties, would be an ideal material for class V restorations [21].

Deep subgingival class V cavities demand special biological properties from a restorative material to be successful in the long-term. The ability of the restorative material to incorporate with both hard tooth tissue and soft periodontal tissues is a major challenge that has not been addressed so far with the currently available restorative systems. There is a need for a restorative material to enable gingival and periodontal re-attachment onto the surface of class V restoration. Development of such properties would significantly improve the survival of deep subgingival restorations by two mechanisms: (1) improving the periodontal health status of the tooth and prevention of further pocket formation and epithelial downgrowth, and (2) establishing a biological soft-tissue seal around the restoration preventing bacterial penetration into the deep subgingival margins of the restoration and development of secondary caries.

Incorporation of bioactive materials to GIC and development of a bio-modified restorative system is proposed in this study to encourage periodontal soft-tissue attachment to the restorative material in deep subgingival cervical cavities. Advanced 3D tissue-engineered gingival mucosal models can be used to assess the interaction of the new bioactive material with the oral soft tissues. The 3D tissue models that we have developed over the past 10 years [22,23] are more clinically relevant and more informative biological test models than the commonly used monolayer cell culture systems and this study will be the first one using these 3D systems for the assessment of the soft tissue interaction with restorative materials. Therefore, the aim of this study was to assess the effects of incorporating bioactive materials to glass ionomer cement (GIC) on its mechanical and biological properties. The hypothesis was that the bio-modification of glass ionomer material would result in the formation of a new restorative material that has acceptable mechanical proprieties and enhanced bioactivity.

## 2. Materials and Methods 

### 2.1. Material Formulation

Six bioactive materials included in this study were:

(a) Hydroxyapatite (CAPTAL, London, UK).
(b) Chitosan (Sigma-Aldrich Company Ltd., Dorset, UK).
(c) Chondroitin sulphate (Sigma-Aldrich Company Ltd., Dorset, UK).
(d) Bioglass (Sigma-Aldrich Company Ltd., Dorset, UK).
(e) Gelatine (Sigma Aldrich Company Ltd., Dorset, UK).
(f) Processed bovine dentin [24], prepared according to the following protocol:

Processed bovine dentin material was fabricated in different stages. Bovine teeth with open apices were extracted and then washed with water and cleaned from all soft tissues including pulp and periodontal ligament. The enamel layer was removed using high-speed diamond bur. Remaining dentine was broken into small pieces (5–10 mm) using a mortar and pestle. The dentine fragments were boiled in distilled water for 2 h to facilitate elimination of any attached soft tissue and cellular elements within dentin and then were refluxed in isopropanol (Sigma-Aldrich Company Ltd., Dorset, UK) for 2 h to remove any remaining soft tissue or fat. After they had been washed with distilled boiling water several times to eliminate the organic solvent, the fragments were air-dried at 100 °C. Final stages involved grinding pieces of dentine into small particle powder using a high-speed mechanical blender, packaging and sterilization by γ-irradiation.

The particle size of the chondroitin sulphate, bioglass, hydroxyapatite was the size provided by the supplier (<100 µm). However, gelatine, bovine dentine and chitosan were either ground manually or by using a ball milling machine. All of the materials were sieved manually with a 45 μm pore size sieve so most of the powder had a particle size of less than 45 µm.

### 2.2. Specimen Fabrication

The bioactive materials were added to the GIC (Fuji IX, GC Corporation, Tokyo, Japan) powder in 10% weight using an electronic bench scale. The addition of the powder was carried out by dry mixing on a paper plate using a plastic spatula until an even distribution was achieved. The liquid was added and mixed with the powder using the same plastic spatula. 

### 2.3. Sample Size

The sample size for the compressive strength was N = 10, based on a previous study by Kumar and co-worker [25], which evaluated the mechanical proprieties of GIC modified with chitosan. To standardize the sample size, 10 samples were prepared from each material for both biaxial flexural strength and hardness tests. Chen and co-worker prepared two samples to measure the setting time after the modification of glass ionomer cement with calcium silicate to enhance its bioactivity [26]. However, in the current study a larger sample size of five samples was prepared from each material to measure working and setting times.

### 2.4. Mechanical Tests

#### 2.4.1. Working and Setting Time Determination

The setting time of the original and modified GIC was measured with the Gillmore needle and a digital timer. A large diameter needle (10 mm) with a weight of 28 g was applied and the working time determined once the needle no longer indents the surface. The sample rotated for the needle to be repeatedly placed on a fresh surface area. This procedure was repeated with a second needle with a smaller diameter (1 mm) and larger weight (400 g) to determine the setting time.

#### 2.4.2. Biaxial Flexural Strength Test

A silicon rubber mould was used to make disc samples with a thickness of 1.2 ± 0.02 mm and diameter of 11.4 mm. After one hour, the specimens were ground with a diamond-embedded media having a nominal grit size of 30–40 µm, and a final polish with media having 15–20 µm diamond grit. The opposing faces of the test samples were checked, as they needed to be flat and parallel. Finally, all specimens were cleaned, and all traces of grinding debris were removed. The discs were prepared at an ambient temperature of 21 °C.

The flexural property was quantified by the BFS test (LLOYD Instrument, LRX 103648, Farham, UK) at a crosshead speed of 1 mm/min. The flexural strength was obtained by measuring the load at fracture in Newton and calculated in MPa with the following equation:

*Smax* = *p*/*h*^2^ [0.606 log_e_ (*a*/*h*) + 1.13],

where

*Smax* = biaxial flexural strength in MPa;

*p* = load in Newton;

*a* = radius of supporting o-ring in mm;

*h* = sample thickness in mm.

This method has been used before to measure the BFS of different glass ceramic materials in two studies [27,28]. 

#### 2.4.3. Vickers Hardness Test

The samples were prepared with the same procedure as those for the BFS test. However, further polishing with two different media having 15–20 µm diamond grit was carried out before performing the tests (Buehler, London, UK). The discs were placed on a Vickers hardness tester (Vickers Armstrong Engineers Ltd., Serial No.: 255002, Grayford, Kent, UK) with a load of 50 g for 10 s. 

#### 2.4.4. Compressive Strength Test

The specimens were prepared in stainless steel moulds (height 6 mm, diameter 4 mm). The internal surfaces of the moulds were evenly coated with petroleum ether prior to filling to facilitate removal of the hardened cement. After one hour, the specimens were immersed in distilled water and kept in an incubator at 37 °C for 24 h. Each specimen was applied between the platens of the mechanical tester and two sheet of damp filter paper were applied both at the top and bottom platens of the test machine (LLOYD Instrument, LRX 103648, Farham, UK) in the area, which will contact the specimens. A fresh piece of paper for each specimen was used. Recording of the maximum force applied when the specimen fractures and the compressive strength (MPa) was calculated using the equation: 

*C* = 4*p*/π*d*^2^,

*p* is the maximum force applied, in newtons;

*d* is the measured diameter of the specimen, in millimetre.

### 2.5. Biological Tests

All materials were purchased from Sigma, UK unless otherwise stated.

#### 2.5.1. Cell Culture

Normal oral fibroblasts (NOF) and TR146 Keratinocytes were obtained from stocks stored in liquid nitrogen at the School of Clinical Dentistry, University of Sheffield. NOFs had been previously obtained from patients having oral surgery with their written informed consent under appropriate research ethics committee approval (National Research Ethics Service, NRES Committee London—Hampstead; Research Ethics Committee (REC) Reference number: 15/LO/0116; date of approval: 21/01/2015). The continuous human cell line TR146 was derived from a neck lymph node metastasis originating from a carcinoma of the buccal oral mucosa. TR146 cells were kindly provided by Cancer Research UK. The cells were cultured in a high glucose complete Dulbecco’s modified eagles medium (CDMEM) supplemented with 10% *v*/*v* foetal bovine serum, 2 mM l-glutamine, 100 U:100 µg mL^−1^ penicillin–streptomycin and 625 ng mL^−1^ amphotericin. All cell types were incubated in a humidified atmosphere at 37 °C and 5% CO_2_/95% air. The medium was changed three times a week until the cells were 90% confluent. 

#### 2.5.2. Investigation of Cell Viability in the Monolayer Culture System

The viability of modified GIC was tested using both NOF and TR146 monolayers. Material specimens were prepared using a silicon rubber mould with a 0.5 mm diameter and 0.4 mm thickness. The samples were sterilized using UV light overnight. The positive control in the experiment was ethanol. Culture medium was used as the negative control. GIC discs without any modification were also tested. From each type of cell, 45 monolayer cell sheets were prepared. The cellular concentration was determined by counting under the light microscope using a manual counter. Fifty-thousand cells were seeded in each well. The medium was changed every other day. The cells were monitored under the light microscope until becoming 90% confluent. The discs were then applied on top of the monolayer and left in the incubator for 24 h.

To monitor the cell viability, Presto-blue viability assay (Invitrogen, Carlsbad, CA, USA) was used. After switching off the light in the cabinet, the old culture medium was aspirated. The monolayers with discs were then washed with PBS twice and 900 µL of new CDMEM was added. One hundred µL PB was then added directly into the well and incubated for 1 h. Three samples of 200 µL were taken and added to 96 well-plates. Then fluorescence reading (560 nm excitation and 590 nm emission) was recorded using spectrophotometric plate reader (Infinite^®^ M200, TECAN, Morrisville, NC, USA). Then, average values were calculated for the five samples.

#### 2.5.3. Investigation Biocompatibility of the Modified GIC Discs Using 3D-OMM Model 

Test specimens were prepared using a silicon rubber mould of 1 cm length and 0.4 mm thickness. The samples were sterilized using UV light overnight. Media was used as the negative control. Glass ionomer discs without any modification were also tested. Three discs were prepared from each material. The samples were fixed in the middle of the tissue culture inserts using orthodontic NiTi wires (Ortho Technology, Palatine, IL, USA).

Fibroblast-populated collagen gel was prepared to engineer the connective tissue layer of the oral mucosal model according to the following method [29]. A solution of DMEM 13.8 mg mL^−1^, FCS 8.5% (*v*/*v*), L-glutamine 2 mM, reconstitution buffer (22 mg mL^−1^ sodium bicarbonate and 20 mM HEPES in 0.062 N NaOH) and 5 mg mL^−1^ rat-tail type I collagen was prepared on ice. Then, neutralization of the solution to reach a pH of 7 was done by adding 1 M of sodium hydroxide solution. A cell suspension made from 200,000 cell of primary fibroblast in CDMEM were added to the previous solution and subsequently added around the test specimens inside the tissue culture inserts. The insert was then left in the incubator at 37 °C for 2 h to solidified and form fibroblast-populated collagen gel. CDMEM media was added inside and outside the insert and incubated for 24 h.

1 × 10^6^ of TR146 cells suspended in 50 µL of the culture medium were seeded on top of the gel surface and allowed to adhere for 2 h. Subsequently, 2 mL of growth medium was gently added into the insert and incubated at 37 °C, 5% CO_2_ for 3 days.

To evaluate cell viability, a PB test was carried out using a similar method as described in the previous section. 

For histological evaluation, 3D human oral mucosa model (3D-OMM) were removed from the inserts, fixed in 10% (*v*/*v*) PBS-buffered formalin for a minimum of 24 h, GIC discs were gently removed and frozen sections were prepared. The OMM was embedded in Optimal Cutting Temperature Compound OCT (Fisher Scientific, Loughborough, UK). Cryosections were then prepared with 10 micrometres thickness. The slices were then mounted onto glass slides. Sections were stained with haematoxylin and eosin using a Leica ST4040 Shandon Linear Stainer (Leica Microsystem) and examined under a light microscope. 

### 2.6. Statistical Analysis

Statistical analysis of the data was carried out with one-way analysis of variance (ANOVA) followed by Tukey multiple comparison test using Minitab statistical analysis software.

## 3. Results

### 3.1. Mechanical Tests

The average values and standard deviations for the results of the mechanical tests are presented in Table 1.

#### 3.1.1. Working and Setting Time

Statistical analysis with one-way ANOVA showed that the addition of bioactive materials to GIC did not have any statistically significant effect on the working and setting times (*p* value > 0.05) except for bioglass, which caused an increase in the working time by approximately 1 min (*p* value < 0.05). 

#### 3.1.2. Biaxial Flexural Strength (BFS) 

After 24 h of the sample storage in the incubator, the control GIC samples’ average BFS value was 33.1 MPa. Modifying GIC by gelatine showed statistically significant reduction in the biaxial flexural strength compared to the control group (*p* value < 0.05). However, all the remaining materials did not reveal in statistically significant difference compared to the GIC discs’ (*p* value > 0.05). 

#### 3.1.3. Vickers Hardness 

The control group GIC showed a 49.5 MPa hardness average value. Statistical analysis showed no significant difference between different test groups and the control group (*p* value > 0.05). 

#### 3.1.4. Compressive Strength 

The average compressive strength value of GIC was 112.3 MPa. Gelatine and chondroitin sulphate showed significant reduction compared to GIC (*p* value < 0.05). GIC samples modified by adding hydroxyapatite, bovine dentine, bioglass and chitosan did not show significant difference compared to GIC group (*p* value > 0.05). 

### 3.2. Biological Tests

#### 3.2.1. NOF Monolayer Viability Assay

**Figure 1:**
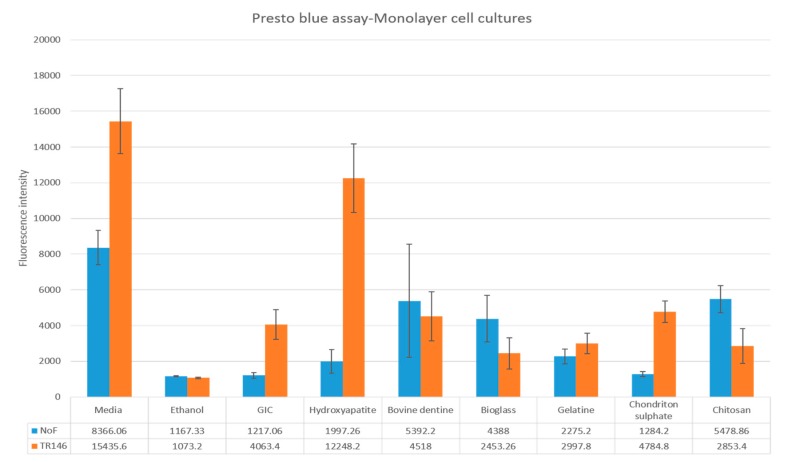
Cell viability of normal oral fibroblasts (NOFs) and TR146 exposed to test materials (10% by weight bioactive material and 90% glass ionomer cement (GIC)) and GIC discs used as a control.

The average values and the standard deviations for the cell viability assays using monolayer cell cultures are represented in Figure 1. According to the Presto-blue results, bovine dentine, bioglass and chitosan caused significant increase in the cell viability of the NOFs exposed to test materials (*p* value < 0.05). Hydroxyapatite, gelatine and chondroitin sulphate did not have any statistically significant effect on the cell viability (*p* value > 0.05).

#### 3.2.2. TR146 Monolayer Viability Assay

Presto-blue results showed only GIC samples modified by hydroxyapatite had a significant increase in the viability of TR146 keratinocytes compared to the control group (*p* value < 0.05; Figure 1). All the other materials had insignificant influence (*p* value > 0.05).

#### 3.2.3. 3D-OMM Model Tissue Viability Assay

**Figure 2:**
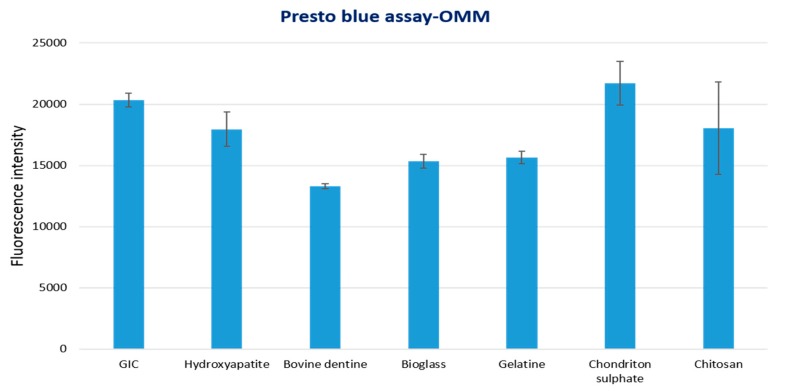
Tissue viability of 3D human oral mucosa model (3D-OMM) exposed to test materials (10% by weight bioactive material and 90% GIC) and GIC discs used as a control.

The results of tissue viability assay for 3D-OMM exposed to test materials are shown in Figure 2. Statistical analysis showed that bovine dentine-, gelatine- and bioglass-modified samples had significantly lower tissue viability compared to control GIC (*p* value < 0.05). The other test groups (hydroxyapatite, chondroitin sulphate and chitosan) showed no statistical significance difference compared to the control group. 

#### 3.2.4. Histological Analysis 

**Figure 3:**
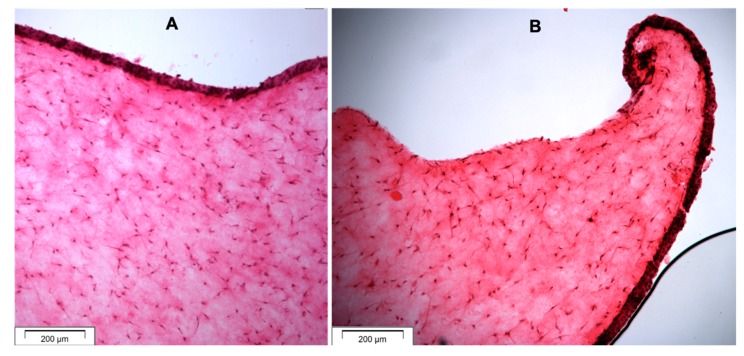
Histological sections (H&E staining) of OMM showing (**A**) the epithelial and connective tissue layers and (**B**) curling of the epithelial layer towards the GIC disc space and forming junctional epithelium.

The histological images of the 3D-OMM are presented in Figure 3. All the histological sections showed curling or growth of the epithelial layer towards the disc space, except for the GIC modified with gelatine (Figure 3).

**Figure 4:**
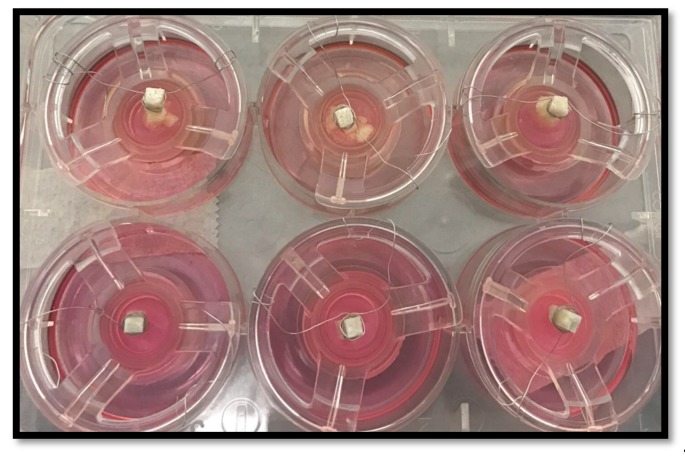
Test samples within 3D oral mucosal model. The upper panel shows the dissolution of discs fabricated from GIC modified with gelatine after 24 h. The lower panel shows stable GIC discs within 3D oral mucosal models.

Figure 4 shows the test samples within 3D oral mucosal models including stable specimens and gelatine-modified GIC discs with evidence of dissolution.

## 4. Discussion

GIC material used in this study was a commercially available self-curing GIC and is presented in powder and liquid format for hand mixing. It is favoured over the encapsulated type of GIC as modification was readily permitted by adding the biological additive to their constituents. In this study, a number of bioactive materials were added to GIC to enhance its biological properties to be used to restore class V lesions. Hydroxyapatite, bioglass, bovine dentine, chitosan, chondroitin sulphate and gelatine were chosen to be added to the powder because of the wide use of these materials in biomedical fields, bioactivity and biocompatibility [30]. This was the first study to investigate the effects of six different bioactive agents on both mechanical and biological properties of the GIC material using advanced 3D tissue-engineered oral mucosal model to mimic the clinical situation as closely as possible.

To identify the behaviour or the proprieties of a material placed in the oral cavity, a variety of mechanical testing can be undertaken. Generally, the response of the material is investigated after force application such as tensile, compressive and shear stresses. The nature of force is usually either static or dynamic. The majority of mechanical testing in dentistry has been carried out in vitro [31]. The main mechanical properties of restorative materials investigated in this study were compressive strength, tensile strength, hardness and working and setting times. 

The bioactive materials that have been used in this study did not affect the working time, except for bioglass, which increased the working time by approximately one minute. On the other hand, no difference was detected in the setting time between the control group and the test groups. The working and setting time proprieties of GIC are influenced by how the cement is prepared, which include the powder to liquid ratio, the concentration of the poly-acid, the particle size of the powder and the specimens age [32].

The ability of the material to bend before it breaks is defined as the flexural strength. When the materials are subjected to high chewing stress, a high flexural strength is desired to prevent permanent deformation. The flexural strength provides a good indication of resistance to tensile failure. Brittle materials such as GIC fail by crack propagation that is caused by tensile force. To measure the tensile strength of a brittle material it is difficult to perform direct tensile strength testing. A study by Prosser et al., have recommended that the flexural strength is the most appropriate measurement method for GIC strength evaluation [31].

The statistical analysis as mentioned previously, showed only a GIC sample modified by gelatine resulted in significant reduction in BFS compared to the control GIC group. The variation in the microstructure and the composition of the newly modified glass ionomer cement may have influenced the strength of the material [33]. The size of the particles may also affect the physical properties of the glass ionomer cement. Reducing the particle size will reduce the voids and improve the strength. In addition, other environmental factors such as the temperature and water may affect the flexural strength of the glass ionomer. In the present study, the samples were immersed in distilled water for 24 h, which may also have affected the mechanical proprieties. The presence of water allows the slow-setting-glass-ionomer to complete the acid-base reaction and minimize crack propagation on the surface.

Vickers hardness is a non-intrinsic propriety of the materials. The test is performed to evaluate the surface characteristics of the material, which are very important to determine, as they can affect the material’s polishing ability. Non-polished restorative material can act as a substrate for plaque accumulation and secondary caries formation. In addition, it affects the material’s ability to resist the loads applied. The hardness value represents the combination of deformation and elastic behaviour. The method used to calculate the hardness value is to measure the depth or area of an indentation left by an indenter, having a specific shape with a specific force at a specific time.

In this study, all the modified materials had a very similar hardness value to control GIC. The reading of indention was extremely difficult in this study due to the indistinct border of the pyramid in the micrograph of the Vickers hardness tester. Yap and co-workers explained this phenomenon as the material pile-up of the material around the indenter due to the material’s plasticity being the main cause of this problem [33]. To extract precise mechanical properties from instrumented indentation, knowing the relationship between indentation load and the true area is crucial. It would be helpful if explicit expressions, relating the true contact area and depth of indenter penetration into the GIC material, are known for different indenter geometries.

Compressive strength is an important feature of GIC to be used as a restorative material, especially in the process of mastication. GIC has a low tolerance when subjected to tension; as such it is important to measure compressive strength. According to the manufacturer, the compressive strength of Fuji IX GIC is 220 MPa. However, there is some variation in the literature regarding the reported mechanical properties of the GIC. In a review study by Yip et al. a range of 130–230 MPa was reported [34]. Bali et al. [35] reported the value of 112 MPa, which is very similar to the findings of our study with regard to the compressive strength of the GIC material. The results discussed previously showed that adding gelatine and chondroitin sulphate to GIC reduced the compressive strength significantly. Both compressive strength and biaxial flexural strength of GIC seems to be reduced by adding gelatine. Gelatine is an organic substance that once incorporated into the GIC may affect its strength.

The influence of hydroxyapatite on the GIC polysalt bridge formation level and its setting reaction may justify maintaining the compressive strength value of the GIC [36]. This concurs with the result from Alatawi et al., where it was found that the addition of hydroxyapatite to the glass ionomer improved its mechanical properties [37]. The addition of bovine dentin to the GIC cement showed similar compressive strength to the control group. The difference between hydroxyapatite and bovine dentin is the presence of the organic group, which may affect the strength. De Caluwé and co-workers [38] showed that adding bioactive glass to the conventional GIC had a similar effect on compressive strength of GIC, which is consistent with the results of this study. However, Kumar et al. found that incorporation of nanochitosan into GIC improved the compressive strength, which was not the case in this study. The reason may have been due to the particle size used. Nanochitosan has a large surface area to react with PAA, which improves the compressive strength of the modified GIC [25].

Restoring a class V cavity is a common procedure to perform in dentistry. However, it is challenging and technically demanding, due to the presence of gingival tissue in close proximity to the restoration. The interface between restoration and gingival tissue should be optimum to ensure proper marginal adaptation. In this study, GIC has been modified by hydroxyapatite, gelatine, bioglass, bovine dentine, chitosan and chondroitin sulphate to enhance attachment with gingival tissue to be used as class V restorative material. This was tested using two systems. The first was the monolayer cell culture system that is fabricated from the main cells of the gingival tissue, which are fibroblasts and keratinocytes. This was followed by investigating the biological properties of the newly modified materials through construction of 3D-OMM models.

In both monolayer and 3D-OMM models, Presto-blue reagent was used to measure cell viability. The Presto-blue reagent has resazurin-based solution that measures the cell viability based on the metabolic activity of the living cells to quantitatively indicate the proliferation of cells through turning the environment to a red colour, becoming highly fluorescent. The changes in colour can be measured using fluorescence and absorbance measurements [39]. Although the standard deviation was significant in some of the groups, statistical analysis showed that the data had a normal distribution and therefore allowed reliable comparison between different groups using one-way ANOVA. 

The GIC modified by bovine dentin showed excellent biocompatibility with NOF and TR146 cells and simulated the proliferation of these cells. This was consistent with the results of a previous study where the bovine dentine was tested for use as a bone substitute [33]. In a study by De Caluwé et al., GIC modified by bioglass showed good biocompatibility to human foreskin fibroblasts, which is in agreement with the result of this study [36]. GIC modified with hydroxyapatite showed similar biocompatibility to control GIC using NOF, but higher biocompatibility using TR146 cells. The monolayer cell culture testing results also confirmed the biocompatibility of the gelatine-modified GIC. Choi et al. [40] and Hong et al. [41] stated that gelatine is a non-antigenic attractant of fibroblast, a macrophage activator and promotes epithelisation and granulation tissue formation. However, the dissolution of the discs fabricated from GIC modified with gelatine within the 3D-OMM model may have prevented the growth of the epithelial layer, as shown in the microscopic structure.

Incorporation of chitosan and chondroitin sulphate did not have any significant adverse effects on the viability of monolayer cell cultures or 3D oral mucosal models in this study. Both chitosan and chondroitin are considered biocompatible materials and have been widely used in scaffolds for oral and dental tissue engineering [22,30]. 

Monolayer cell cultures of fibroblasts were more sensitive to dental materials tested in this study compared to the 3D oral mucosal models. This could be one of the limitations of the monolayer cell cultures for biological evaluation of dental materials and the results obtained from monolayer systems may not be extrapolated to the clinical situation.

The 3D tissue-engineered oral mucosal model used in this study has been shown previously to be a clinically relevant in vitro test system that would reduce the need for animal testing and be more specific and has the potential to be used as a suitable biocompatibility test model for investigating biological effects of different dental biomaterials and oral care products [12,22,23]. 

## 5. Conclusions

The results of this study indicate that hydroxyapatite, chitosan, bioglass and processed bovine dentine can be incorporated into GIC without compromising the mechanical properties. Addition of processed bovine dentine, bioglass and chitosan increase the cell viability of the normal oral fibroblast monolayers exposed to the GIC specimens. Biomodification with hydroxyapatite can significantly increase the viability of the exposed monolayers of TR146 keratinocytes. However, bovine dentine, gelatine and bioglass may have an inhibitory effect on the tissue viability of the 3D oral mucosal model tested in this study.

## Figures and Tables

**Table 1:** Mean values and standard deviations of the results of the mechanical tests for different test groups. * Represents statistically significant difference from the control group (*p* < 0.05).

	Working Time (Minutes)	Setting Time (Minutes)	BFS (MPa)	Hardness (MPa)	Compressive Strength (MPa)
**GIC**	5.1 (0.5)	1.6 (0.5)	33.1 (6.8)	49.5 (6.8)	112.3 (17.1)
**Hydroxyapatite**	6.6 (0.5)	1.6 (0.4)	31.4 (5.1)	52.6 (13.1)	129.5 (26.5)
**Bovine dentine**	5.9 (0.5)	1.7 (0.3)	31.0 (4.0)	47.9 (13.9)	111.3 (25.9)
**Bioglass**	6.7 * (1.2)	2.0 (0.3)	32.0 (2.1)	46.3 (15.6)	91.0 (14.9)
**Gelatine**	6.0 (0.3)	2.4 (0.5)	21.8 (6.3)	41.4 (18.7)	44.2 * (16.6)
**Chondroitin sulphate**	6.2 (0.7)	2.2 (0.8)	35.0 (4.4)	37.5 (11.8)	50.5 * (10.8)
**Chitosan**	4.9 (0.3)	1.2 (0.3)	35.0 (4.4)	49.1 (23.3)	97.1 (14.7)
